# Cell-free synthesis of a functional G protein-coupled receptor complexed with nanometer scale bilayer discs

**DOI:** 10.1186/1472-6750-11-57

**Published:** 2011-05-23

**Authors:** Jian-Ping Yang, Tatiana Cirico, Federico Katzen, Todd C Peterson, Wieslaw Kudlicki

**Affiliations:** 1 Life Technologies, 5791 Van Allen Way, Carlsbad, CA 92008, USA

## Abstract

**Background:**

G protein coupled receptors (GPCRs) represent the largest family of membrane proteins in the human genome and the richest source of targets for the pharmaceutical industry. A major limitation to characterizing GPCRs has been the difficulty in developing high-level heterologous expression systems that are cost effective. Reasons for these difficulties include inefficient transport and insertion in the plasma membrane and cytotoxicity. Additionally, GPCR purification requires detergents, which have a negative effect on receptor yields and stability.

**Results:**

Here we report a detergent-free cell-free protein expression-based method to obtain pharmacologically active GPCRs in about 2 hours. Our strategy relies on the co-translational insertion of modified GPCRs into nanometer-sized planar membranes. As a model we employed an engineered β2-adrenergic receptor in which the third intracellular loop has been replaced with T4 lysozyme (β2AR -T4L). We demonstrated that nanolipoprotein particles (NLPs) are necessary for expression of active β2AR -T4L in cell-free systems. The binding specificity of the NLP- β2AR-T4L complex has been determined by competitive assays. Our results demonstrate that β2AR-T4L synthesized *in vitro *depends on similar oxidative conditions as those required by an *in vivo*-expressed receptor.

**Conclusions:**

Although the activation of β2AR-T4L requires the insertion of the T4 lysozyme sequence and the yield of that active protein limited, our results conceptually prove that cell-free protein expression could be used as a fast approach to express these valuable and notoriously difficult-to-express proteins.

## Background

With over 1000 members, G-protein-coupled receptors represent the largest family of integral membrane proteins, whose corresponding genes span > 1% of the human genome [1]. Despite their remarkable topological similarity (they all have a seven-transmembrane -α-helical topology), they respond to a plethora of extracellular stimuli, mediating cellular responses to hormones, neurotransmitters and senses such as smell, taste, and sight. Their relevance in signal transduction and their extraordinary diversity have turned them into the most pursued drug targets (> 60% of the industry), which generate $50 billion in pharmaceutical sales per year [2].

Major limitations to studying GPCRs include the difficulties in using high-level heterologous expression platforms (being baculoviral-mediated and transient mammalian expression the most commonly used) and the unpredictability of the expression outcome. It is apparent that the folding mechanism and stability of these molecules in their native systems are quite complex. This notion imposes a key obstacle, as the natural abundance of these proteins is too low to purify sufficient material for functional and structural studies.

Another difficulty is that GPCRs are naturally embedded in a complex and dynamic lipid bilayer, which restricts the use of many standard biophysical techniques used for studying soluble proteins. The use of detergents is often questioned, as it is unclear how well micelle structures mimic the natural membrane protein environment. On the other hand, reconstitution of GPCRs into artificial bilayers is not a trivial process, and results in structures that are too heterodisperse to be used in structural studies.

Another complication in the study of GPCRs is their inherent conformational plasticity. With the exception of rhodopsin, which is locked into an inactive state by its covalently bound inverse agonist 11-*cys*-retinal, GPCRs adopt different states of activity, which precludes determining their tridimensional structure [3]. Recent breakthroughs have been the resolution of ligand bound inactive-state structures of GPCRs [4-9], where a variety of protein modifications and engineering approaches were applied to stabilize the proteins.

Cell-free protein expression is increasingly being considered as viable alternative for overcoming the above obstacles (for a recent review see [10]). However, all current cell-free approaches used to express functional GPCRs require protein solubilization in detergent micelles or reconstitution into lipid membranes [11-14]. In order to circumvent these obstacles, we have recently proposed a novel cell-free protein expression strategy based on the use of 'nanodiscs' or 'nanolipoprotein particles' (NLPs) [15,16].

NLPs are self-assembled discoidal particles composed of a planar phospholipid membrane bilayer surrounded by an apolipoprotein ring (scaffold protein) [17]. The approach makes use of NLPs, which are either added to or *in situ *assembled in the cell-free reaction, providing support for the nascent membrane protein and thereby circumventing subsequent protein extraction and reconstitution steps. The main limitation is that wild-type GPCRs expressed under these conditions lack ligand binding activity (unpublished results).

Here we report the cell-free synthesis of functional human adrenergic β2 receptor (β2AR) that has been stabilized through the insertion of the T4 bacteriophage lysozyme (β2AR-T4L) [5]. We also show that ligand binding activity is attainable without the need of detergent solubilization or membrane reconstitution.

## Results

### β2AR-T4L complexes with NLPs and binds a specific antagonist - relevance of the lysozyme insertion

**Figure 1:**
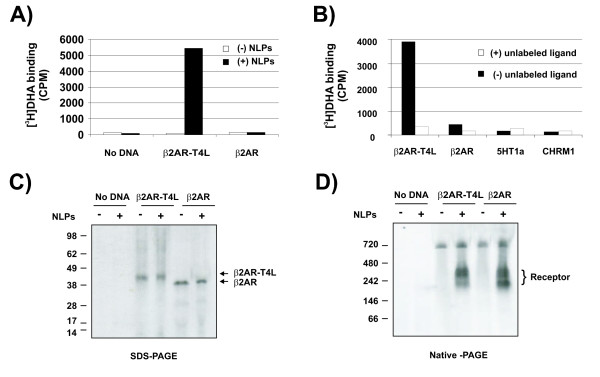
**Antagonist binding of the β2AR-T4L-NLP complex**. A) Cell-free protein expression was performed in the presence of 40 nM [^3^H]DHA. The unbound isotope was removed by affinity purification and binding was determined as described in methods. B) Similar reactions as in A) were performed in the presence of NLPs with or without excess of unlabeled propranolol. Two unrelated GPCR proteins 5HT1a and CHRM1 were used as negative controls. C) Similar reactions as in A) were performed in the presence of 1.5 mM [^35^S]Met. Proteins were separated by SDS-PAGE and visualized by autoradiography. D) Products from the reaction described in C) were separated by native electrophoresis and visualized by autoradiography. Bands with an apparent MW of 720 Kd are non-specific. Experiments in (A)-(D) were performed using the MembraneMax Protein Expression Kit (Life Technologies, Carlsbad, CA) employing DTT-free buffers with the indicated modifications (see methods).

In an effort to produce functional GPCRs *in vitro *without relying on detergent solubilization or membrane reconstitution, nine members of this receptor family were expressed in the presence of NLPs [15]. Unfortunately, none of them, albeit associated with NLPs, exhibited ligand binding activity (results not shown). After numerous unsuccessful attempts that involved the reworking of the expression conditions, the addition of folding catalysts, and the use of a variety of N- and C- terminal fusion partners, we focused our attention on a recently reported stabilized variant of β2AR [5,6]. The molecule harbors a T4 lysozyme insertion within the third intracellular loop, which does not impair critical biochemical consequences on the receptor other than slightly elevated agonist binding affinities [5,6]. When this protein was expressed in our cell-free protein expression system in the presence of NLPs, specific and displaceable binding to the antagonist [^3^H]-dihydroalprenolol ([^3^H]DHA) was observed (Figure 1A and 1B). In contrast, β2AR failed to exhibit binding even though identical reaction conditions were applied (Figure 1A and 1B). Additional experiments showed that other non-specific ligands failed to bind the NLP- β2AR-T4L complex (not shown). The receptor variants (β2AR-T4L and β2AR) expressed at similar levels (Figure 1C), and complexed with the NLPs in a similar fashion, generating a supramolecular complex of an apparent weight of about 250 kDa (Figure 1D). However, only β2AR-T4L was able to bind [^3^H]DHA (Figure 1A). Previous reports indicated that cholesterol was relevant in the stability and function of β2AR [18,19]. However, in our conditions, the addition of cholesterol to the reaction did not result in significant changes in the activity of *in vitro *expressed β2AR or β2AR-T4L (not shown). Taken together, these results suggest that the insertion of the T4 lysozyme moiety (major difference between the two receptor variants) may play a crucial role assisting the molecule to attain a functional and/or more stable conformation in preformed NLPs.

### Pharmacological characterization of cell-free expressed β2AR-T4L

**Figure 2:**
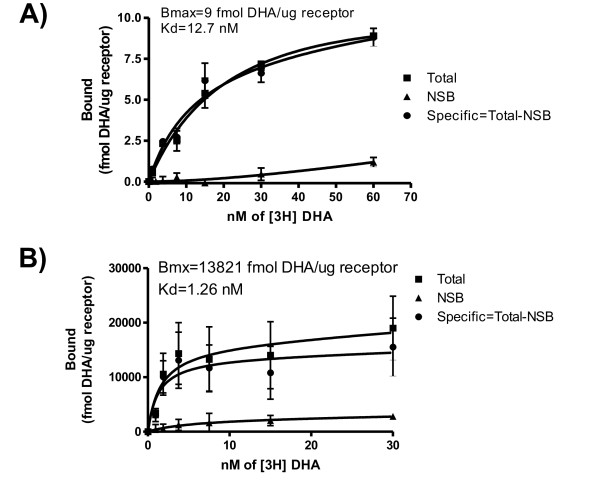
**Functional characterization of β2AR-T4L**. (A) The protein was expressed *in vitro *and ligand binding was assessed on crude material as described in the text. Protein yield was estimated by trace-labeling with [^35^S]Met. As a control, purified receptor expressed in insect cells [5] was used. (B). Binding assays for (A) and (B) were performed as described in methods [5]. Propranolol was used as the unlabeled competitor. Experiments in (A) were performed using The MembraneMax Protein Expression Kit (Life Technologies, Carlsbad, CA) using DTT-free buffers with the indicated modifications (see methods). Kd and Bmax values were calculated using GraphPad PRISM software (GraphPad Software, San Diego, CA). Abbreviations: NSB, non-specific binding;

In order to assess whether β2AR-T4L was properly folded, we determined its ligand binding constants. Our approach combines cell-free protein expression and ligand binding in a single reaction. The radioligand is added at time zero, and binding takes place as proteins are translated and complexed with the NLPs. Protein concentration and folding is driven by the amount of programming DNA and reaction conditions. Therefore, higher variability than in conventional ligand binding assays is expected. Saturable affinity binding curves were obtained using [^3^H]DHA as the radioligand, and propranolol as the unlabelled competitor. Results showed that the dissociation constant (Kd) of the cell-free product (Figure 2A) was close to what is observed with β2AR-T4L expressed and purified from insect cells (Figure 2B), However, the estimated maximal number of receptor binding sites (Bmax) for the cell-free made protein was significantly lower than that one produced in insect cells (compare Figure 2A with 2B). These findings, as will be discussed below, suggest that only a small fraction of the total protein produced attained its proper active conformation.

One way to estimate the amount of active receptor produced stems from the calculated Bmax parameter (9 fmol DHA/μg receptor). Assuming one binding site per molecule (MW = 56941 Da), and knowing that the overall GPCR yield (according to [^35^S]Met trace labeling) was 100 μg/ml, 51 ng of GPCR/ml of reaction was obtained. A second approach to estimate that value is through the equation "Bmax cell-free expressed receptor/Bmax insect cell-expressed receptor × yield cell-free expressed receptor", assuming the insect-cells expressed protein is fully functional. Applying the latter formula the total amount of active receptor per ml of reaction should be 65 ng/ml, very close to the number above. Differences inherent to the expression platform used (insect cells vs. prokaryote cell-free) such as post-translational modifications, membrane composition, and sample processing may also account for this discrepancy.

**Figure 3:**
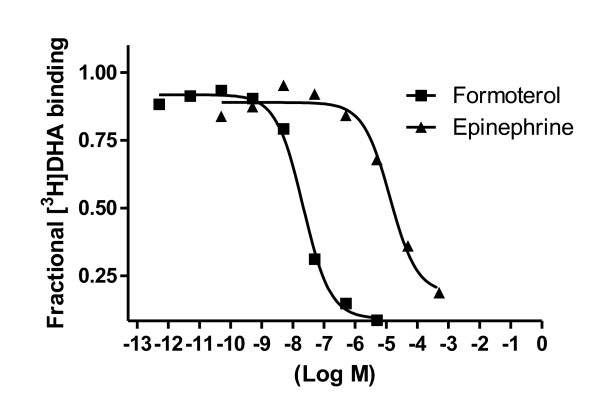
**Affinity competition curves for adrenergic ligands**. Binding experiments were performed on crude material from cell-free reactions as described in methods using [^3^H]DHA and the indicated unlabelled competitors.

To further characterize the cell-free expressed receptor, affinity competition experiments were carried out. The natural antagonist epinephrine and the high-affinity synthetic agonist formoterol were used (Figure 3). The relative affinities for these ligands was similar as that one observed using membranes isolated from insect cells expressing β2AR-T4L [5].

Taken together, the results above suggest the protein synthesized *in vitro *exhibit similar pharmacological properties as those described earlier [5]. These findings validate the cell-free protein expression approach as a viable vehicle to obtain functional β2AR-T4L, albeit with limited folding capacity.

### Oxidative protein folding is required for ligand binding

**Figure 4:**
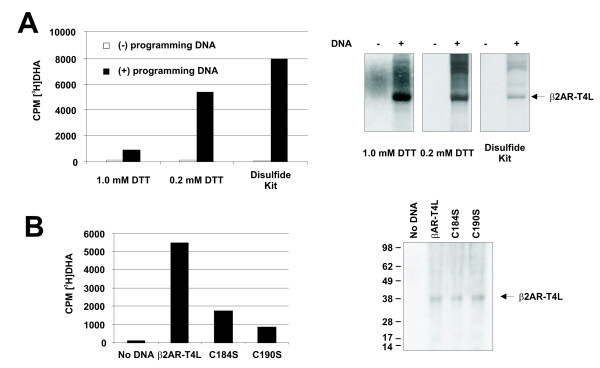
**Disulfide bond requirements for β2AR-T4L**. (A). Ligand binding assays for β2AR-T4L expressed *in vitro *was performed as described in Fig. 1 using the MembraneMax Protein Expression system (Life Technologies, Carlsbad, CA) following the manufacturer's directions, which results in 1.0 mM DTT in the reaction; or using DTT-free buffers, which results in 0.2 mM DTT in the reaction; or using the RTS 100 *E. coli *Disulfide Kit (Roche Applied Science, Indianapolis, IN), which is designed for synthesis of disulfide-bonded proteins (detailed composition unknown). All reactions were performed in the presence of NLPs and either 0.5 nM ^[3^H]DHA (for ligand binding determination) or [^35^S]Met (for protein quantity assessment). [^35^S]Met trace-labeled products were separated by SDS electrophoresis, and visualized by autoradiography). (B) Reactions using β2AR-T4L and two cysteine mutant derivatives were performed using the MembraneMax Protein Expression system supplemented with DTT-free buffers and processed as described in (A).

Early reports showed that two extracellular disulfide bonds in the wild type β2AR are required for ligand binding [20,21]. More recently, it was shown that the ligand accessibility in β2AR-T4L is enabled by these pairs of closely spaced disulfide bridges [6]. To verify that the activity of the *in vitro *expressed β2AR-T4L still relies on disulfide bond formation, we expressed this protein using cell-free expression systems with different reducing strengths. The results showed that increasing reducing potential is detrimental to the ligand binding capacity of β2AR-T4L (Figure 4A). In addition derivatives of this protein where two of the essential cysteines were replaced by serine residues produced receptors with significantly lower specific binding activity (Figure 4B).

In summary, our results demonstrate that β2AR-T4L synthesized *in vitro*, depends on similar oxidative conditions as those required by an *in vivo *expressed protein.

## Discussion

The primary objective of our work was to find a practical solution to the limitations of GPCR expression imposed by heterologous systems. Although relatively large amounts of membrane proteins can be potentially produced in cellular systems, usually a small proportion becomes associated to the membrane [22]. Particularly, GPCRs are confronted to a complex array of trafficking signals, post-translational modifications, and transport systems before reaching the final destination, the plasma membrane. In addition, differences in the lipid bilayer composition and maximal tolerated membrane protein loads can additionally affect the correct insertion, folding, and yield of recombinant GPCRs.

In order to overcome these difficulties, we developed a cell-free expression system supplemented with planar membranes [15]. Although, the approach excels in expressing soluble membrane protein products, it fails to produce functional GPCRs. We favor the absence of a functional translocon machinery embedded in the membrane particles as one of the major reasons for this negative outcome.

We reasoned that a way to overcome this deficiency would be to employ GPCR derivatives that insert into the membrane in a translocase-independent fashion (for a recent review on membrane protein insertion see [23]). Since there is no *in silico *approach to establish *a priori *the type membrane insertion mechanism of a given protein, our strategy was to try active variants reported in the literature. Our attempt was to employ one of the stable β2AR derivatives recently described, where the third intracellular loop was replaced by T4 lysozyme [6].

The strategy proved to work. The enzyme complexed with the NLPs and exhibited similar affinity properties as those ones reported *in vivo *[5]. Perhaps the clearest advantage of this method is its short processing time: it only takes about 2 hours to go from gene to ligand binding assay.

Although the cell-free made enzyme showed a similar dissociation constant as that one made *in-vivo*, the maximal number of ligand sites appeared to be significantly lower. Namely, only a fraction of the receptors seems to be active. These findings suggest that additional components such as folding catalysts, specific lipid constituents, post-translational modifications, or a membrane protein insertion machinery, absent in our reaction conditions, may be required by β2AR-T4L to attain a high specific activity. Conversely, elimination of some proteases present in the cell-free extract may contribute to provide a better context for more stable GPCRs. While this observation does not affect the benefit of this approach as a rapid screening method for analyzing GPCR activity, further experimentation is needed to establish the nature of the deficiency. For example, the generation of cholesterol-containing NLPs, or the use of NLPs bearing the SEC translocon would likely shed light on this subject (for reconstitution of the SecYEG complex into NLPs see [24]).

The replacement of the third intracellular loop by T4 lysozyme as a strategy to stabilize GPCR molecules has been proven successful for other GPCR molecules [9]. In addition, other GPCR stabilization approaches have demonstrated success (see for example [8]). Finally, the incorporation of unnatural amino acids into crucial regions of these elusive molecules holds the promise of overcoming some of these difficulties [25].The assay of these other variants in our cell-free approach will help determine what aspects of the GPCR biochemistry are critical for reaching its proper conformation.

## Conclusion

Our results conceptually prove that cell-free protein expression could be used as a fast approach to express these valuable and notoriously difficult-to-express proteins. To our knowledge, this was the first time that a functional GPCR was obtained in a cell-free context, with no further re-folding or reconstitution requirements. The modified cell-free system combined with NLP offers the unique opportunity to produce sufficient expression levels of soluble and functional folded GPCR for pharmacological assays that are difficult to obtain with other expression systems. Finally, the use of NLPs with different chemistries should allow fine-tuning of the conditions optimal for expression and solubilization of specific MPs in near-native context.

## Methods

### Plasmids and clones

The wild type version of the human adrenergic β2 receptor (β2AR, GenBank acc# NM_000024), human serotonin receptor 1a (5HT1a, GenBank acc# NM_000524), human muscarinic 1 receptor (CHRM1, GenBank acc# NM_000738), were retrieved from the Ultimate ORF collection (Life Technologies, Carlsbad, CA), PCR-amplified, and cloned into the plasmid pEXP5-NT/TOPO (Life Technologies, Carlsbad, CA). Details of the construct that expresses β2AR-T4L were published elsewhere [5]. Site directed mutagenesis of the cysteine residues was performed utilizing the GENEART site directed mutagenesis kit (Life Technologies, Carlsbad, CA) employing the following oligonucleotide pairs: 5'-CATCAGGAAGCGATCAACTCCTATGCGGAAGAAAC-3' and 5'-GTTTCTTCCGCATAGGAGTTGATCGCTTCCTGATG--3' (for cysteine 184), and 5'-GGAAGAAACTTCCTGCGACTTTTTCACCAACCAGGCG-3' and 5-CGCCTGGTTGGTGAAAAAGTCGCAGGAAGTTTCTTCC-3' (for cysteine 190).

### Cell-Free Protein Expression Reactions

Cell-free protein expression was conducted using the MembraneMax™ Protein Expression Kit (Life Technologies, Carlsbad, CA) either following the manufacturer's direction or replacing the buffers with similar ones devoid of DTT. Where indicated the RTS 100 *E. coli *Disulfide Kit (Roche Applied Science, Indianapolis, IN) was used in a batch configuration supplementing the reactions with NLPs as indicated previously [15]. Where indicated, reactions were supplemented with [^35^S]Met (135 mCi/mmol final) (Perkin Elmer, Waltham, MA) or varying amounts of [^3^H]DHA (Perkin Elmer, Walthman, MA) as indicated.

### Protein yield and ligand binding determination assays

Yield in cell-free protein expression reactions was determined by [^35^S]Met trace labeling. Reaction products were separated by SDS-PAGE or by native electrophoresis using NativePAGE™ Novex^® ^4-16% Bis-Tris Gels (Life Technologies, Carlsbad, CA) and visualized by autoradiography.

Binding assays were performed in the presence of [^3^H]DHA added directly to the cell free protein expression reactions. Non-specific binding was assessed by performing identical reactions in the presence of 1 μM propranolol (Sigma-Aldrich, Saint Louis, MO) as indicated. Unbound isotopes, [^35^S]Met and[^3^H]DHA, were removed by purifying the protein products through Ni^+2 ^affinity beads (Life Technologies, Carlsbad, CA) via a histidine tag fused to the receptor molecule. Briefly, the binding assay was performed on a 96-well GF/C filter plate (Perkin Elmer, Walthman, MA), pre-treated with 0.3% polyethylenimine (PEI), and washed four times with 500 μl of ice cold binding buffer (50 mM Tris-HCl, pH7.4, 150 mM NaCl, 5 mM imidazole). Then 100 μl of nickel resin beads were added and washed 5 times with 150 μl of binding buffer. Beads were then suspended in 55 μl of binding buffer and mixed with 45 μl of cell-free reaction samples for 20 min at RT. The binding reaction was stopped by washing 5 times with 150 μl of binding buffer. After air-drying, the filters were introduced into scintillation vials, mixed with 3 ml scintillation liquid and measured for bound [^3^H]DHA with a Beckman LS5000 scintillation counter. The binding assay for insect protein samples was conducted as described [5]. Competition binding reactions were carried out in the presence of 0.5 nM [^3^H]DHA plus increasing concentrations of unlabelled formoterol (MP Biomedicals, Solon, OH), or epinephrine (USP, Rockville, MD) and processed as described above. Raw data was processed and analyzed using software package GraphPad Prism (GraphPad Software, San Diego, CA).

## Abbreviations

GPCR: G-protein-coupled receptor; NLP: Nanolipoprotein particle; β_2 _AR: adrenergic β_2 _receptor; 5HT1a: serotonin receptor 1a; CHRM1: muscarinic 1 receptor; DHA: dihydroalprenolol; DTT: dithiothreitol.

## Competing interests

The authors declare that they have no competing interests.

## Authors' contributions

JPY, FK, and TK conceived the work. JPY performed most of the experiments and wrote the manuscript draft. TC performed some of the ligand binding assays. FK wrote the manuscript and helped to analyze the data. TP and WK supervised the whole work. All authors read and approved the final manuscript.
